# An analytical toolbox to verify the presence, quantity and origin of recycled cotton fibres in textile garments

**DOI:** 10.1038/s41598-026-39268-y

**Published:** 2026-02-13

**Authors:** Anke B. G. M. Ten Berge, Robin Temmink, Maud Kuppen, Maaike B. van Slagmaat, Sven Kamphuis, Richard A. J. Groeneveld, Anton Luiken, Jens Oelerich, Jan W. G. Mahy

**Affiliations:** 1 Research Group Sustainable & Functional Textiles, Saxion University of Applied Sciences, Van Galenstraat 19, 7511 JL Enschede, The Netherlands; 2 Alcon Advies B.V., De Aa 31, Wierden, 7642 HA The Netherlands

**Keywords:** Analytical chemistry, Polymer chemistry, Characterization and analytical techniques

## Abstract

The textile industry is one of the most polluting industries. To reduce its environmental impact, the entire supply chain must transition from a linear to a circular economy, making textile recycling at end-of-life critical. Stimulated by new regulations, the industry is increasingly incorporating recycled – mainly mechanically – cotton fibres into new garments. However, producing garments with recycled fibres is costlier than using virgin fibres. Currently, there is no independent way to assess the quantity of mechanically recycled cotton claimed by producers. As a result, “greenwashing” is frequently encountered. In this study, an analytical toolbox was developed to enable independent verification of the claimed mechanically recycled cotton content in garments. For the toolbox three analysis methods were developed. Microscopy can be used to qualitatively confirm the presence of mechanically recycled cotton fibres. Measuring the fibre length distribution allows the amount of recycled fibres to be determined semi-quantitatively. Degree of polymerisation (DP) measurements can be used to distinguish the pre- and/or post-consumer origin of the recycled fibres. Once further enriched and developed into a standard, the toolbox will be an asset in reducing greenwashing and boosting the use of recycled fibres.

## Introduction

The textile industry has grown substantially over the years and remains heavily dependent on non-renewable resources. At the same time, the accelerated disposal of the textile products has led to a rapidly increasing volume of textile waste^1^. The European Union acknowledges this problem by identifying textiles as one of the main industries for the Circular Economy Action Plan^2^. This plan outlines key measures to transition towards a circular textile industry in Europe. This includes enhancing transparency through implementing the Digital Product Passport (DPP) and stimulating textile recycling via the Extended Producer Responsibility (EPR)^3^. By increasing traceability and transparency across the textile supply chain, these measures encourage sustainable practices and support informed decisions regarding the optimal end-of-life scenario for each product^4^.

Textile waste streams are generally divided into three categories: post-industrial, pre-consumer and post-consumer waste. The first two refer to textiles that have not been used by consumers. Post-industrial waste is created during the production process, while pre-consumer waste consists of finished garments that were not sold for various reasons. In this study, both post-industrial and pre-consumer waste are collectively referred to as pre-consumer waste. The third category, post-consumer waste, includes textiles that have been used by consumers, either for commercial purposes such as workwear or for personal use^5^.

In the textile industry, three main recycling methods are available: mechanical, chemical and thermo-mechanical recycling. Thermo-mechanical recycling is limited to synthetic (thermoplastic) materials and is highly sensitive to contaminants such as non-meltable materials and finishes. Compared to mechanical recycling the fibre quality is relatively good^5^. Chemical recycling can be used for synthetic and natural materials, each material requires a specific procedure. Chemical recycling produces high quality fibres, but the process often involves the use of (toxic) solvents such as carbon disulfide or N-methyl-morpholine-N-oxide (NMMO)^6^. Chemical recycling technologies are not yet scalable for major fibre feedstocks like polyester and cotton and are therefore not widely implemented in the market. Mechanical recycling, in contrast, tears textiles into fibres through mechanical action. This method has been established for many years and is currently the predominant technique used in the industry. A key disadvantage is the reduction in fibre quality^2^, primarily due to shortening of the fibre length caused by mechanical forces during processing. As a result of their different origins, differences in fibre quality also exist between pre- and post-consumer mechanically recycled fibres. Pre-consumer materials, having not been extensively used, generally undergo fewer treatments such as laundering and drying, and experience less mechanical wear, resulting in lower fibre degradation, i.e. the quality of the material before recycling is higher than that of post-consumer materials^7^. Consequently, pre-consumer fibres retain greater length during mechanical recycling than post-consumer fibres^8,9^. Additionally, pre-consumer recycled fibres generally show a lower short fibre content and higher fibre strength. These properties are crucial for further processing, making pre-consumer recycled fibres more desirable to yarn producers. In some cases the type of recycled fibres used is not clearly communicated^10^.

In the context of sustainability, industry practices rely primarily on certification schemes. However, the fragmentation and length of supply chains create conditions that enable fraudulent activities. The European Commission highlights the importance of addressing greenwashing: 53% of all green claims appear to provide ambiguous, misleading or unsubstantiated information, whether intentionally or unintentionally^11^. In addition, 50% of all green labels lack independent and transparent verification^12^. Consequently, there is an increasing need to trace the types of fibres used in textile products^13^. To support this, the digital product passport is increasingly being implemented. The DPP can also incorporate the use of tracers, such as chemical tags. With precise dosing devices and reliable tracking systems the recycled fibre content could be quantified. However, this does require the addition of tracer material, often in the form of fibres containing chemical components^4^. DPPs are important developments, as they are considered enablers of a circular economy^14^. Currently a wide variety of product passports exist, differing in scope and application, and there is no common agreement of which specific data should be included. However, there is a lack of common understanding what specific data should be included^15^. In addition, DPPs often rely on the integrity of the supply chain partners providing the input data. To further reduce the risk of greenwashing, it is essential to analyse and verify the recycled (cotton) content and its origin in post-consumer textiles. Adding verified results to DPPs will strengthen the technology. Currently, no methods are available to determine the recycled cotton content in garments. Research on textile (fibre) analysis is dominated by methods based on (near)infrared (NIR) spectroscopy, usually applied for sorting textile waste to promote recycling^16,17^. Infrared-based spectroscopy is also the method of choice for forensic purposes, where textile fibre identification often provides crucial evidence^18^. Mäkelä et al. use NIR to distinguish between virgin cotton and regenerated cellulose, such as lyocell and viscose, based on their polymer chain length (degree of polymerisation, DP) and crystallinity^19^. Mahlamäki et al. further developed the method to estimate DP classes of 100% cotton materials for chemical recycling^20^. However, none of these methods currently address the detection of recycled content.

This study aimed to design an analytical toolbox to identify, quantify and determine the origin of mechanically recycled cotton fibres in polycotton textile garments. Three analysis methods were evaluated for their ability to distinguishing (post-consumer) recycled cotton fibres from virgin fibres. *Microscopy* was used to examine fibre ends for signs of mechanical recycling like cutting, tearing and shredding. *Fibre length distribution (FLD)* measurements assessed whether the amount of shorter mechanically recycled fibres could be determined semi-quantitatively. *Degree of polymerisation* (DP) analysis was used to evaluate the potential to distinguishing *post*- from *pre-*consumer textiles. The methods were applied to various polycotton textiles of known composition and to industry garments to validate sustainability claims.

Section 2 describes the methods comprising the toolbox, while Sect. 3 details the textile materials used. Section 4 presents and discusses the experimental results. Section 5 provides a summary and outlook of the findings.

## Methods

The three methods developed in this study – microscopy, fibre length distribution and degree of polymerisation – are in detail described in this section.

### Microscopy

**Fig. 1:**
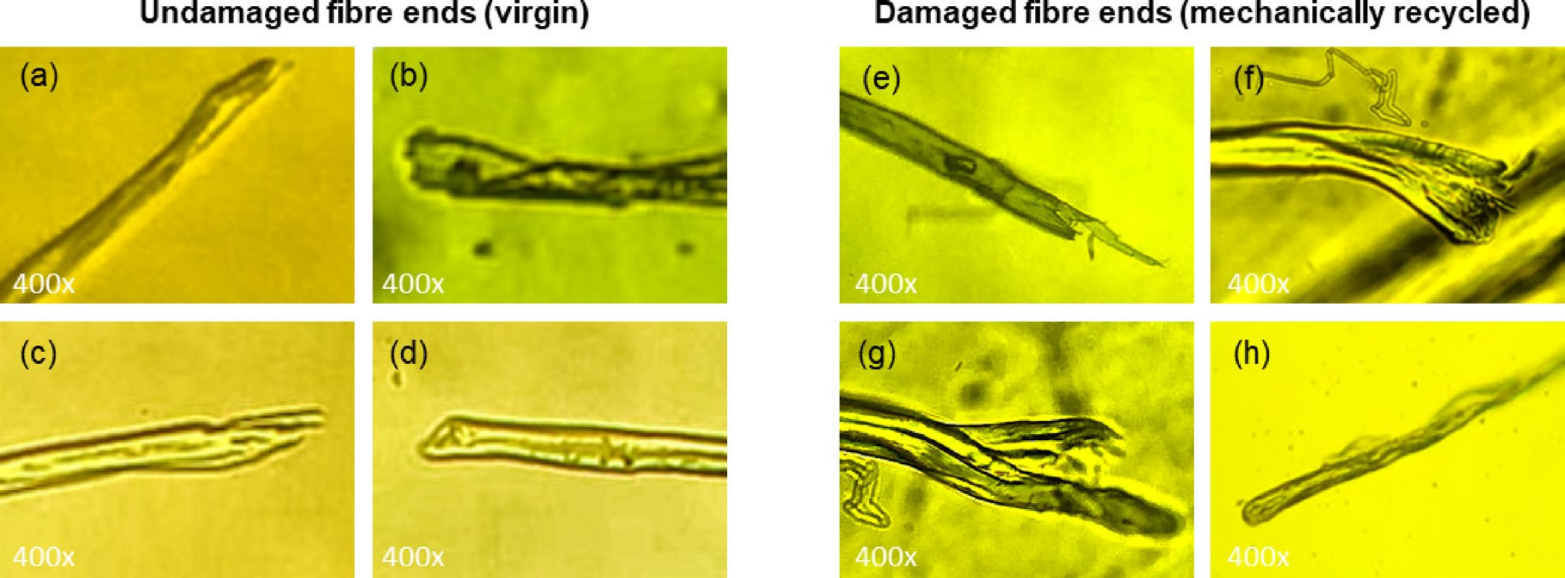
Examples of natural, undamaged fibre ends from virgin cotton fibres **(a-d)** and damaged fibre ends from mechanically recycled cotton fibres **(e-h)**.

Yarns were carefully untwisted by hand to allow observation of individual fibres from the centre of the yarn. The untwisted yarns were examined under an optical microscope equipped with a 10x/18 ocular and 4x/0.65, 10x/0.65 and 40x/0.65 objective lenses. Cotton fibres were identified based on their appearance (Sect. 4.1). Cotton fibre ends were imaged at 400x magnification by photographing through the ocular. For each yarn, 100 cotton fibre ends were imaged and analysed for signs of mechanical damage. Four blind raters independently classified the fibre ends as ‘**D**’ (damaged*)*, ‘**U**’, (undamaged) and ‘**A**’ (ambiguous). Distinctions between ‘**D’** and ‘**U’** were guided by a set of exemplary images, a selection of which is shown in Fig. 1. For fibres classified as **A**, raters additionally provided a best guess of ‘**D’** or ‘**U’.** Only images for which the raters agreed (allowing a maximum of one initial ‘**A**’ rating) were selected for interpretation.

### Fibre length distribution

**Fig. 2:**
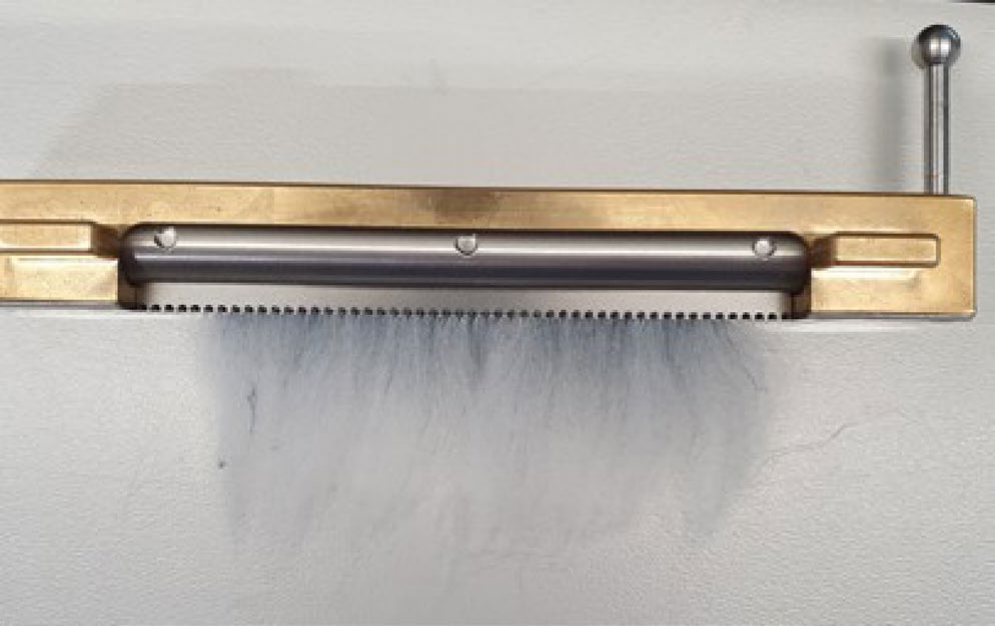
Photograph of the fibre magazine of the FIBROTEST. One sample is estimated to contain between 12,000 and 25,000 individual fibres (equipment supplier information).

Yarns were manually removed from the fabrics to obtain single fibres for measurement. To facilitate dissection and minimise fibre breakage, the yarns were first partially untwisted using a twist tester. Individual fibres were then carefully extracted from the yarn core using a fine pin. For each yarn, 1 gram of fibres was collected and placed in the sample preparation unit of the FIBROTEST. The base of the sample preparation unit was filled using a textile cloth, pushing the fibres through the openings allowing collection by the fibre magazine (Fig. 2). Fibre length measurements were performed according to ASTM D1447-07^21^ using the Textechno FIBROTEST. The same 1 gram of fibres for each yearn was prepared and measured 10 times.

To retrieve the composition of the yarns *semi-quantitatively*, the following data processing steps were applied. Raw data was smoothed with a moving average of 5 and transformed into frequency tables. The number of fibre types expected in each yarn was identified based on microscopy results – if available – and label information (claimed compositions), and subsequently confirmed by discerning distinct distributions in the frequency distribution plots.

The overall frequency plot was then subdivided into the distributions of the individual fibre types. This was performed by manual assessment, assuming Gaussian distributions for natural and recycled natural fibres and left-skewed normal distributions for man-made fibres (see Sect. 4.2). Typically, the shorter-fibre distribution can be attributed to mechanically recycled fibres, while the longer-fibre distribution corresponds to man-made fibres. Virgin cotton fibres, when present, generally peak between those two ranges.

Because textile compositions and sustainability claims are expressed in weight percentages, *frequency* distributions were converted into *weight* distributions. This was done by multiplying the total fibre length of each fibre type by its linear density (Table 1). The final composition was calculated as the relative weight contribution of each distribution.

Additionally, the relative proportions of the fibre type distributions can be cross-checked against the cellulose content determined from the fibre ratio analysis^22^ (see following section) to improve accuracy. In this study however, this step was not performed in order to specifically assess the suitability and accuracy of the fibre length distribution method on its own.

Results are reported in weight percentages. Deviations are expressed as absolute differences, since the goal was to evaluate the absolute deviation in composition, which should be as small as possible.

**Table 1:** Linear density of the fibres used in this study (Table 2). The linear densities of fibre 3 and fibre 5 were provided by the suppliers. The average linear densities ($\:{\rho\:}_{l,\:avg})$ of fibre 1 and fibre 2 were calculated using $\:{\rho\:}_{l,\:avg}={p}_{cot}\:\times\:{\rho\:}_{l,\:cot}+{p}_{pet}\:\times\:{\rho\:}_{l,pet}$, where $\:{p}_{cot}$ and $\:{p}_{pet}$ represent the fractions of cotton and polyester, respectively, as determined from fibre ratio tests. $\:{\rho\:}_{l,\:cot}$ and $\:{\rho\:}_{l,\:pet}$ denote the linear densities of cotton (Fibre 4) and polyester (Fibre 5), respectively. For the proof of principle presented in this study, values of $\:{\rho\:}_{l,\:cot}$ for Virgin cotton (Fibre 4) as well as pre- and post-consumer mechanically recycled cotton were taken from Arafat and Uddin (2022)^7^.

Fibre	Linear density
Fibre 1	1.68 dtex
Fibre 2	1.69 dtex
Fibre 3	1.7 dtex
Fibre 4	1.58 dtex
Fibre 5	1.95 dtex

### Degree of polymerisation

Although nearinfrared-based approaches for DP assessment have been reported^19,20^, these require specialized instrumentation and advanced ICT expertise to develop appropriate models and algorithms. For the present toolbox, viscosity-based DP measurements were selected, as they are standardized and widely used. The degree of polymerisation was determined by measuring the intrinsic viscosity of a solution of cellulose dissolved in an alkaline iron-tartaric sodiumcomplex (EWNN), following ISO 5351 − 2^23^ and DIN 54270–3^24^. While the standard method applies to 100% cotton, in this study it was extended to analyse the cellulose fraction in *polycotton blends*.

For each textile material (Sect. 3), one or more independent 5 g aliquots were prepared by milling using a power cutting mill equipped with a 1 mm sieve. Each aliquot of textile material is reported as a separate sample (e.g., Sample 1, 2, …). The samples were stored in a climate chamber set to 20 °C and 65% relative humidity for at least 24 h. For each sample the cellulosic content was determined using the fibre ratio analysis method according to ISO 1833-11:2017^22^. EWNN solution was prepared according to DIN 54270–3^24^, stored refrigerated and used within one week. In a 25.00 mL volumetric flask, the sample was weighed using an analytical balance to obtain a cellulose fraction between 7 and 9 mg per replicate. Next, 10–15 mL of EWNN solution was added, followed by thorough shaking. The flask was then placed in the refrigerator overnight under continuous shaking at 300 rpm. The flask was subsequently filled to 25.00 mL with EWNN solution and homogenized by manually inverting the flask ten times.

For polycotton samples, the solution was transferred into a 50 mL centrifuge tube and centrifuged at 4000 rpm for 20 min at 15 °C to compact the polyester at the bottom. Approximately 20 mL of the clear cellulose-EWNN solution was collected using a 10 mL Finnpipet.

Intrinsic viscosity $\:\left[\eta\right]$ was determined by measuring the lead time of the solution using an Ubbelohde (type 530 13) placed in a thermostatic bath at 20 °C, equipped with a viscopump and viscosity measuring system. For each solution the measurement was repeated three times and the deviation allowed was ± 0.1 s. For each sample, two separate solutions were prepared and analysed (duplo), with a maximum allowed deviation of 5% between duplicates.

The intrinsic viscosity $\:\left[\eta\right]$ is calculated using the Schulz-Blaschke equation: $\:\left[\eta\right]=\frac{\left({\eta}_{rel}-1\right)/c}{1+{k}_{\eta}\left({\eta}_{rel}-1\right)}$, with $\:c$ the cellulose concentration (g/mL), $\:{k}_{\eta}$ = 0.339 and $\:{\eta}_{rel}=\frac{t}{{t}_{0}}$; with $\:t\:$and $\:{t}_{0}$ the lead times of the cellulose solution and pure EWNN solvent in seconds, respectively. The molar mass $\:{M}_{polymer}$ was calculated using the Mark-Houwink equation: $\:\left[\eta\:\right]=K\times\:{{M}_{polymer}}^{a}$ with $\:K\:$= 1.72 × 10^− 3^ and $\:a$ = 1,01 for cellulose in EWNN. Finally, the DP was calculated as: $\:DP=\frac{{M}_{polymer}}{{M}_{monomer}}$ with $\:{M}_{monomer}$ the weight of the cellobiose monomer (342.3 g/mol^−1^).

To verify that the presence of polyester during cellulose dissolution in EWNN does not influence the measured cellulose DP, control experiments were performed. A standard cotton reference (CN-11, bleached woven cotton, Center for Test Materials, B.V.) was mixed with polyester fibres (Fibre 5, Table 2) in weight ratios ranging from 34% cotton/66% polyester to 67% cotton/33% polyester. These mixtures were prepared and analysed following the procedure described above. The resulting cellulose DP values were compared to those obtained for the cotton reference alone. No significant differences were observed ($\:{DP}_{cot,blank}\:$= 1926 ± 23; $\:{DP}_{cot,mix}$= 1937 ± 24), indicating that the presence of polyester does not affect the viscosity-based DP measurement of cellulose in polycotton blends.

## Textile materials

To validate the analytical methods, a selection of textile materials was used (Table 2). Textiles of known composition were produced on laboratory-scale equipment. Hereto, fibres were selected to represent those commonly used in (sustainable) textile products: mechanically recycled cotton of pre- and post-consumer origin (Fibre 1 and 2), man-made cellulose (Fibre 3), (raw) virgin cotton (Fibre 4) and polyester fibres (Fibre 5). The batches of mechanically recycled fibres (Fibre 1, Fibre 2) contain small amounts of non-cellulosic material, predominantly polyester. With fibre ratio analysis, performed according to ISO 1833-11:2017^22^, the cellulosic content was determined to be 86.7% (std 0.7) for Fibre 1 and 95.5% (std 2.8) for Fibre 2.

From these fibres, five yarns (Yarn 1–5) were produced. The fibre blends were selected to encompass a range of material combinations and complexities while ensuring the yarns were sufficiently strong for lab-scale weaving. Fibre opening and alignment were performed using a Mesdan Felt Carder 337 A. Each batch was carded three times to ensure proper opening, mixing, and alignment. After the third pass, the fibres were fed through the coiler to form slivers. Fibre losses during carding were determined for the blended yarns (Yarns 1–4) and amounted to 3.4%, 3.7%, 5.1%, and 4.2%, respectively. As a result, the final yarn composition may slightly differ from the intended input listed in Table 2, since relatively more recycled and natural fibres were lost compared to man-made fibres due to their shorter length. Drawing was carried out on the Mesdan Stirolab 3371, further aligning the fibres and adjusting the sliver to the target roving thickness of approximately 4 ktex. The rovings were then spun into yarns using a Schlafhorst Oerlikon Autocoro 480 rotor spinning machine, resulting in yarns with a yarn count of ~ 70 tex and a twist of ~ 400 tpm. Finally, two fabrics (Fabric 1 and Fabric 2) were woven in a plain weave on a CCI Evergeen 500 weaving loom.

In addition, two commercial fabrics (Fabric 3 and 4) were supplied by industry partners. Label information was provided, as well as the confirmation that the fabrics were produced using mechanically recycled cotton fibres. Further production details were not provided.

For each test method, textile materials were selected based on availability.

**Table 2:** Textile materials. Fibres 1–4, yarns 1–5 and fabrics 1–2 were produced in-house. fabrics 3–4 were provided by industry partners.

Name	Description	Composition (claimed)
Fibre 1	Pre-consumer mechanically recycled denim	-
Fibre 2	Post-consumer mechanically recycled cotton knitwear	High cotton content
Fibre 3	Lyocell RB	≥ 30% recycled cotton from pre- and postconsumer waste ≤ 70% wood pulp
Fibre 4	Raw virgin cotton	Raw organic cotton
Fibre 5	Virgin polyester	Virgin polyester
Yarn 1	Polycotton yarn with pre-consumer recycled denim	30% Fibre 1 70% Fibre 5
Yarn 2	Polycotton yarn with post-consumer recycled knitwear	60% Fibre 2 40% Fibre 5
Yarn 3	Cellulose yarn with pre-consumer recycled denim and lyocell.	33% Fibre 1 67% Fibre 3
Yarn 4	Polycotton yarn with post-consumer recycled knitwear and raw virgin cotton	33% Fibre 2 33% Fibre 5 34% Fibre 4
Yarn 5	Virgin cotton yarn	100% Fibre 4
Fabric 1	Woven polycotton fabric with pre- and post-consumer mechanically recycled fibres	Warp: Yarn 1 (~ 50%) Weft: Yarn 2 (~ 50%)
Fabric 2	Woven polycotton fabric	Warp: Yarn 3 (~ 50%) Weft: Yarn 4 (~ 50%)
Fabric 3	Khaki polycotton woven garment for the workwear industry	50% recycled polyester 50% recycled textile – cotton rich – of pre- and post-consumer origin.
Fabric 4	White polycotton terry cloth for the professional industry	48% recycled cotton 52% recycled polyester

## Results & discussion

### Microscopy

Optical microscopy was used to *qualitatively* confirm the presence of mechanically recycled cotton fibres. During the mechanical recycling process, fibres are retrieved from the fabric by cutting, shredding and opening, which causes characteristic fibre-end damage. These types of damage can be distinguished microscopically from the natural fibre ends of virgin fibres(Fig. 1). Undamaged fibre ends look natural (Fig. 1a-d), whereas mechanically recycled fibres typically show *frayed* or *fragmented* fibre ends (Fig. 1e-g) caused by shredding and opening, or *sharp cut* ends, looking like a straight edge, (Fig. 1h) resulting from cutting.

Since fibre damage also occurs during textile production processes (e.g., carding and spinning), a comparison was made between a 100% virgin cotton yarn (Yarn 5, Fibre 4) and a polycotton yarn containing post-consumer mechanically recycled cotton and polyester (Yarn 2, Fibre 2 and 5, respectively). This analysis aimed to verify whether the microscopy method could distinguish recycled from virgin cotton based on the fraction of damaged fibre ends.

For each yarn, 100 fibre-ends were imaged, randomized, and blindly rated by four independent raters who where unaware of yarn type and composition. Of the total 200 images, 61 images (32 from Yarn 2 and 29 from Yarn 5) were consistently rated and included for analysis. The limited number reflects the exploratory nature of the study: some images were difficult to interpret due to fibre orientation or focus, while others require further research to conclusively classify them as damaged or undamaged.

**Fig. 3:**
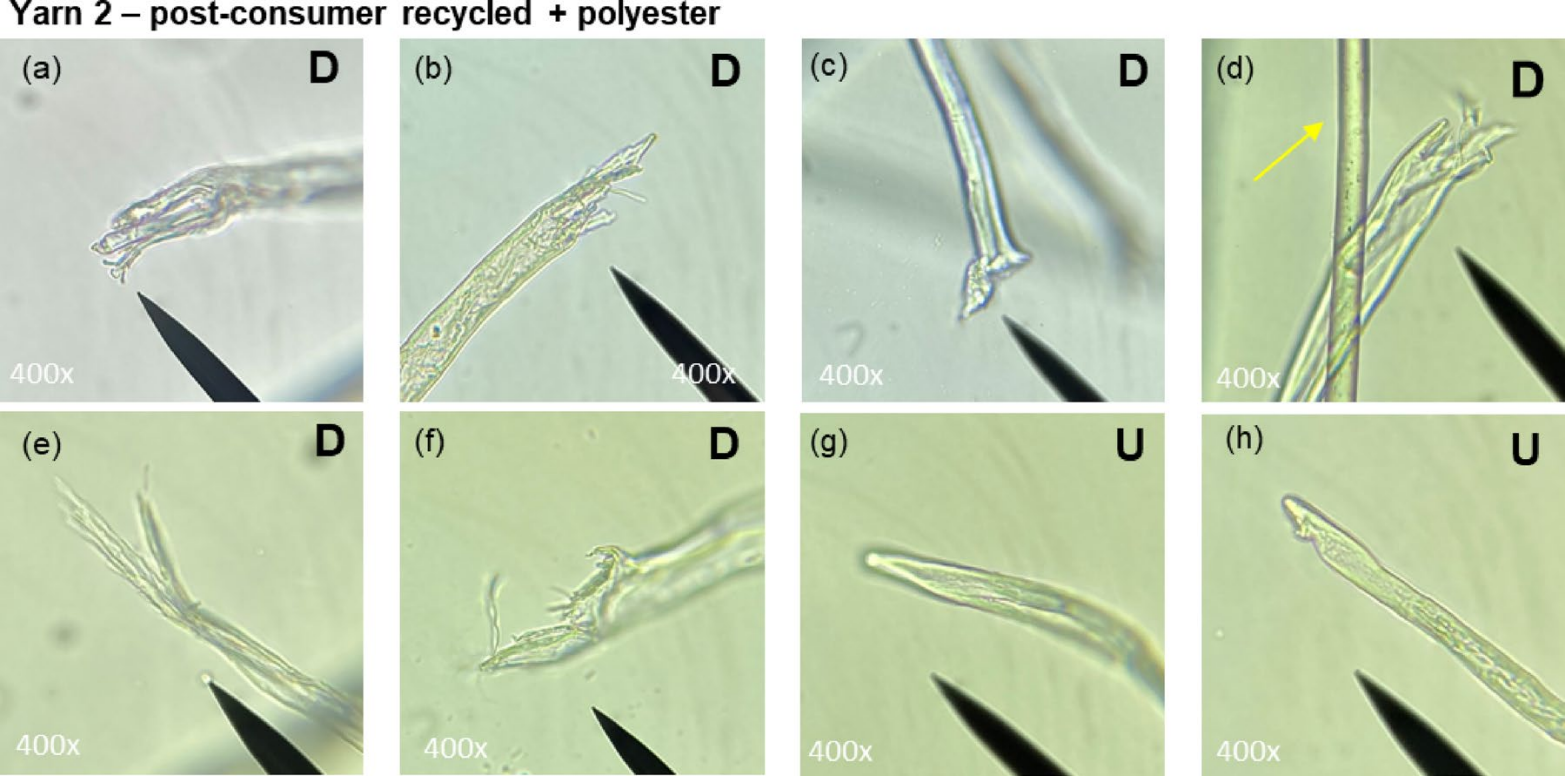
Microscopy images of damaged ‘**D**’ **(a-f)** and undamaged ‘**U**’ **(g-h)** cotton fibre ends from Yarn 2. The yellow arrow illustrates a man-made fibre (polyester).

**Fig. 4:**
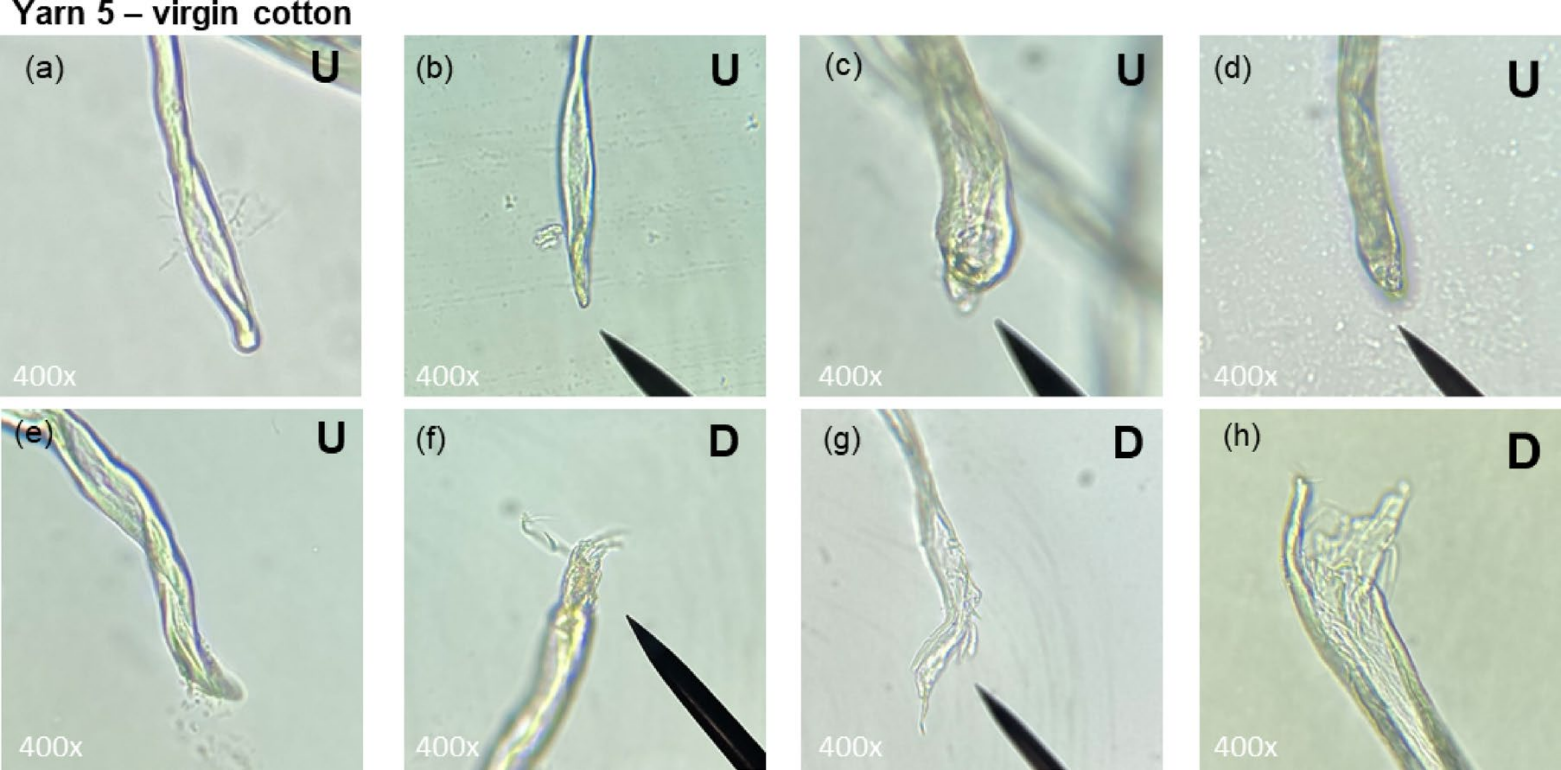
Microscopy images of undamaged ‘**U**’ **(a-e)** and damaged ‘**D**’ **(f-h)** cotton fibre ends from Yarn 5.

Figures 3 and 4 show representative images of Yarn 2 and Yarn 5, respectively. Both yarns contained damaged and undamaged fibre ends. Undamaged fibre ends appeared smooth and natural (Figs. 3g-h and 4a-e). Damaged fibre ends mostly appeared frayed (Figs. 3a, b and d-f and 4f-h). Sharp, cut fibre ends – typical for cutting during recycling – were observed only in Yarn 2 (Fig. 3c). Additionally, man-made polyester fibres could be easily distinguished from cotton fibres by their smooth surface (Fig. 3d), whereas cotton fibres display distinct surface irregularities.

**Fig. 5:**
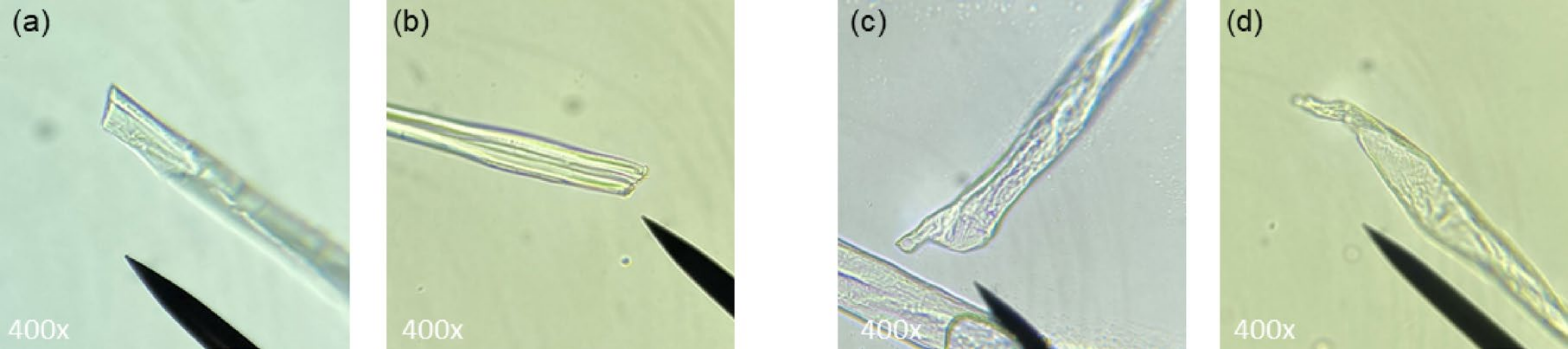
Examples of microscopy images excluded from analysis because they were not consistently classified. **(a**,** b)** presumably show sharp, cut fibre ends from Yarn 2 (recycled cotton), while **(c**,** d)** show presumed natural, undamaged fibre ends from Yarn 5 (virgin cotton).

Table 3 summarizes the microscopy results. For Yarn 2 (mechanically recycled cotton) most of the fibre ends (91%) were classified as damaged, while only a limited amount of undamaged fibres (9%) were present. In contrast, for Yarn 5 (virgin cotton) the fraction of damaged fibre ends is considerably lower (66%) while the proportion of undamaged fibres was significantly higher (34%). The fraction of *undamaged* fibres is likely underestimated, as the full range of natural fibre-end appearances in virgin cotton has yet to be established, which may have led to conservative classification by the raters, especially in Yarn 5. Similarly, due to the exploratory nature of the research, sharp, cut fibre ends were not yet well defined and were therefore not always rated identically. As a result, for the *recycled* yarn (Yarn 2), the *damaged* fraction may have been slightly underestimated. Representative images of such unselected fibre ends are displayed in Fig. 5.

**Table 3:** Results of microscopy analysis of yarn 2 and 5.

	Yarn 2 Recycled cotton	Yarn 5 Virgin cotton
Fraction of damaged fibre ends (‘D’)	91%	66%
Fraction of undamaged fibre ends (‘**U’)**	9%	34%
Number of fibre ends included for analysis	32	29

The results confirm that microscopy can *qualitatively* identify the presence of mechanically recycled cotton fibres, based on the fraction of fibre-end damage present. However, since both virgin and recycled fibres exhibit some degree of damage, and not all fibre ends can be conclusively categorized, the method is unsuitable for quantitative determination. Future work is required to establish reference ranges for damaged fibre fractions in virgin versus recycled textiles. To this end, systematic imaging of fibres at different processing stages, both virgin and mechanically recycled, will be performed. Machine-learning-based automated image classification will be used to support the development of clear image-acquisition and classification guidelines and automated damage quantification. Additionally, further research will investigate whether the presence of sharp cut fibre ends is specific to recycled yarns and could serve as a definitive indicator of mechanically recycled content.

### Fibre length distribution

In mechanical recycling processes, fabrics are cut and torn into smaller pieces, yarns and ultimately fibres. This mechanical action shortens the fibre length, allowing distinction between shorter mechanically recycled fibres and longer virgin cotton or man-made fibres. Based on this principle, the fibre length distribution method (Sect. 2) was developed to semi-quantitatively determine the composition of textile materials.

**Fig. 6:**
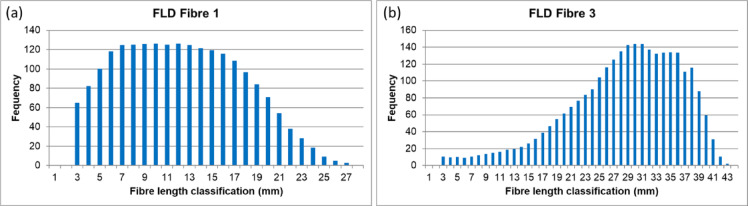
Fibre length distribution of **(a)** Fibre 1 (natural recycled) and **(b)** Fibre 3 (man-made).

Before applying the method to the fabrics, the fibre length distributions of the various fibre types used in this study (Fibres 1–5) were measured as benchmark. Two illustrative examples – Fibre 1 (natural, mechanically recycled) and Fibre 3 (man-made, lyocell RB) – are shown in Fig. 6. In the literature, cotton fibre length distributions have been described using both Weibull functions^25,26^ and Gaussian distributions^27,28^. In this proof-of-principle study, Gaussian distributions were chosen for reasons of simplicity and alignment with the method, which currently contains a human assessment component (Sect. 2).

The measured frequency distributions of the natural fibres followed a Gaussian profile (Fig. 6a), albeit with a left-side truncation attributed to the detection limit of the measurement device. The frequency distributions of man-made fibres (Fig. 6b) could be described by a left-skewed Gaussian distribution, resulting from man-made fibres being cut at a specific length during production.

**Fig. 7:**
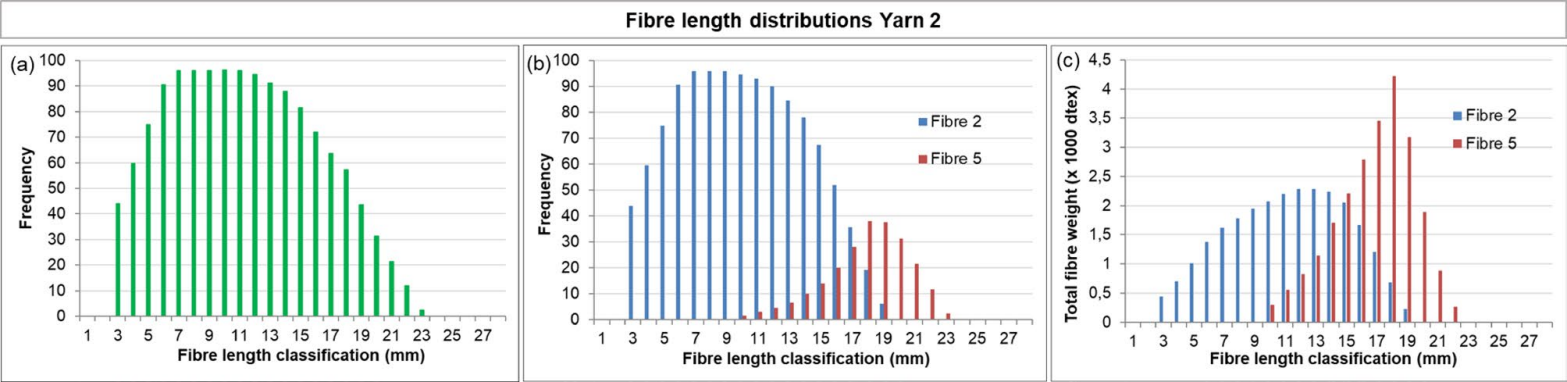
Fibre length distribution of Yarn 2 (post-consumer recycled knitwear, Fibre 2 + polyester, Fibre 5), retrieved from Fabric 1. **(a)** Bimodal fibre length distribution of the yarn, estimated **(b)** frequency distributions for Fibre 2 and Fibre 5 **(c)** weight distributions for Fibre 2 and Fibre 5.

**Fig. 8:**
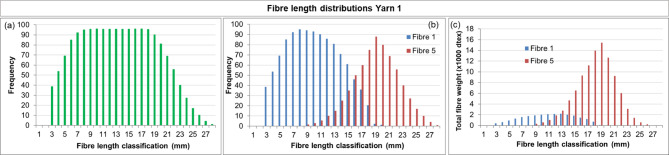
Fibre length distribution of Yarn 1 (pre-consumer recycled denim, Fibre 1 + polyester, Fibre 5), retrieved from Fabric 1. **(a)** Bimodal fibre length distribution of the yarn, estimated **(b)** frequency distributions for Fibre 1 and Fibre 5 **(c)** weight distributions for Fibre 1 and Fibre 5.

The fibre length distribution method was then applied to fabrics with known compositions (Fabric 1 and Fabric 2, Table 2) to validate whether their compositions could be retrieved semi-quantitatively. The results for the yarns retrieved from Fabric 1 are shown in Figs. 7 and 8.

Figure 7a shows the fibre length distribution for Yarn 2, which consists of mechanically recycled fibre (post-consumer, Fibre 2) and polyester (Fibre 5). The distribution is positively skewed supporting a bimodal distribution with peaks near 8 mm and 17–18 mm, indicating the presence of two fibre types with overlapping length ranges. Microscopy results (Sect. 4.1) further corroborates this interpretation by revealing the presence of mechanically recycled cotton fibres as well as man-made fibres (polyester). The peak near 8 mm can be attributed to the shorter mechanically recycled cotton fibres, whereas the peak at higher lengths corresponds to the longer polyester fibres. The overall distribution in Fig. 7a was decomposed into two component *frequency* distributions (Fig. 7b), which were subsequently converted into *weight* distributions (Fig. 7c) following the method described in Sect. 2. For the man-made fibres, the resulting distribution shows slightly reduced left-skewness compared to the input (Fig. 6b), likely due to fibre damage during manual preparation, when fibres were removed from the yarn.

In a similar manner, the results of Yarn 1, also consisting of mechanically recycled fibre (pre-consumer, Fibre 1) and polyester (Fibre 5), retrieved from Fabric 1, are shown in Fig. 8. Here, the known composition was used to support bimodality. When the full toolbox is employed, microscopy will instead provide this supporting information, as demonstrated for Yarn 2 above. Comparing Figs. 7 and 8 highlights a clear distinction: Yarn 2 contains a higher fraction of mechanically recycled fibres. This is reflected in the weight distributions, where Fig. 7c shows a larger recycled fraction than Fig. 8c, consistent with expectations based on the input materials (Table 2).

The compositions of both yarns were estimated from the weight distributions and compared to the input proportions (Table 4). For Yarn 1, the estimated composition was 20% mechanically recycled cotton and 80% polyester, underestimating the recycled cotton content by 10% points. For Yarn 2 the mechanically recycled content was estimated at 53%, compared to ~ 60% input, an underestimation of 7% points. Consequently, the man-made fibre content was overestimated by 10 and 7% points for Yarn 1 and Yarn 2, respectively. These deviations illustrate the semi-quantitative nature of the method.

**Fig. 9:**
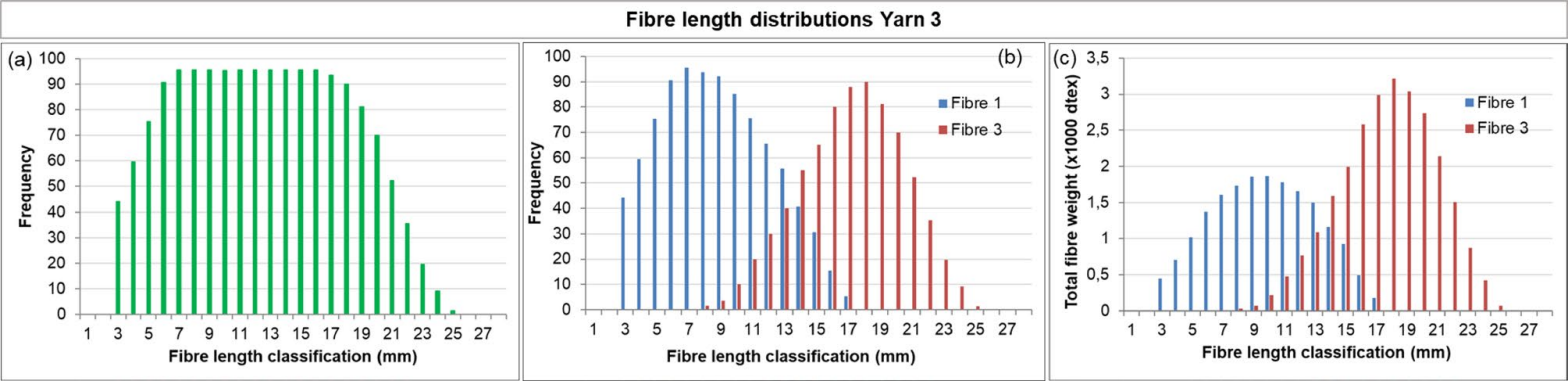
Fibre length distribution of Yarn 3 (pre-consumer mechanically recycled denim, Fibre 1 + lyocell RB, Fibre 3), retrieved from Fabric 1. **(a)** Bimodal fibre length distribution of the yarn, estimated **(b)** frequency distributions for Fibre 1 and Fibre 3 **(c)** weight distributions for Fibre 1 and Fibre 3.

**Fig. 10:**
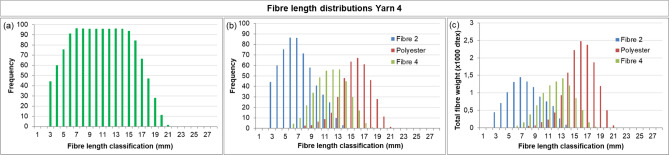
Fibre length distribution of Yarn 4 (post-consumer recycled knitwear, Fibre 2 + virgin cotton, Fibre 4 + polyester, Fibre 5), retrieved from Fabric 1. **(a)** Bimodal fibre length distribution of the yarn, estimated **(b)** frequency distributions for Fibre 2, 4 and 5 **(c)** weight distributions for Fibre 2, 4 and 5.

The results for Fabric 2 are presented in Fig. 9 (Yarn 3) and Fig. 10 (Yarn 4). Yarn 3 is a two-component blend of mechanically recycled fibre (pre-consumer, Fibre 1) and man-made fibre (Fibre 3, lyocell RB). Its nominal composition is similar to Yarn 1, with ~ 30% mechanically recycled fibres and ~ 70% man-made fibre. However, comparison of the results for Yarn 3 (Fig. 9) with Yarn 1 (Fig. 8), reveals differences. These are most evident in the weight distributions, which are influenced by fibre linear density: polyester has a considerably higher linear density than lyocell RB (Table 1). Comparing Figs. 8c and 9c shows that Yarn 3 appears to contain a higher fraction of mechanically recycled fibres. This is reflected in the estimated compositions (Table 4): for Yarn 3, the mechanically recycled content (Fibre 1) is *overestimated* by 8% points, whereas for Yarn 1 it was *underestimated* by 10% points. These deviations further highlight the semi-quantitative character of the method.

The results of Yarn 4 (from Fabric 2) are shown in Fig. 10a. This yarn is more complex due to the presence of three fibre types – mechanically recycled cotton (post-consumer, Fibre 2), virgin cotton (Fibre 4), and polyester (Fibre 5) – resulting in greater uncertainty in interpretation. For the untrained observer, only two discrete distributions might be distinguished. When the full toolbox is employed, microscopy can reveal the presence of man-made fibre as well as a mix of damaged and undamaged fibre ends with a ratio suggesting three fibre types, although microscopy alone would likely not be sufficient to draw definitive conclusions. For such complex yarns, label information is essential. With the label indicating three fibre types, the frequency distribution can be subdivided into three individual frequency and weight distributions (Fig. 10b, c).The estimated composition is presented in Table 4, with deviations comparable to those observed for the other yarns.

Overall, these results demonstrate that the fibre length distribution method could be validated on the fabrics with known compositions. The method can semi-quantitatively estimate the amount of mechanically recycled content present, enabling verification of claimed compositions within a tolerance of approximately ± 10% points. This deviation primarily reflects the inherent error of the method but can partly be attributed to fibre loss during spinning, where shorter fibres are preferentially lost (Sect. 3).

Accuracy can be further improved by incorporating the cellulosic content determined from fibre ratio analysis. It should be noted that this method does not provide definitive identification of fibre types or the origin of the recycled content; such confirmation requires complimentary methods from the toolbox, such as microscopy and DP determination. Future development will focus on implementing automated fitting algorithms to eliminate human bias and on optimising and automating the labour-intensive sample preparation step.

**Table 4:** Estimated compositions of the yarns retrieved from fabric 1 and fabric 2 compared to the input compositions (Table 2).

	Yarn 1	Yarn 2	Yarn 3	Yarn 4
Estimated content mechanically recycled fibres (%)	20	53	41	29
Input mechanically recycled fibres (%)	30	60	33	33
**Absolute difference (%)**	**−10**	**−7**	**8**	**−4**
Estimated content man-made fibre (%)	80	47	59	41
Input man-made fibre (%)	70	40	67	33
**Absolute difference (%)**	**10**	**7**	**−8**	**8**
Estimated content Fibre 4 (%)	-	-	-	29
Input Fibre 4 (%)	-	-	-	34
**Absolute difference (%)**	**-**	**-**	**-**	**−4**

### Degree of polymerisation

The degree of polymerisation (DP) of cellulose, the building block of cotton fibres, reflects the average chain length of the polymer and can be used as an indicator of material history. In particular, DP is relevant to differentiate between pre- and post-consumer textiles, as processing, laundering, and use reduce the degree of polymerisation primarily through alkaline hydrolysis and oxidative degradation that cause chain scissions^29^. Within the toolbox, DP analysis thus serves as a means to distinguish pre- and post-consumer recycled fibres.

To enable distinguishment between pre- and post-consumer recycled fibres, it is essential to establish which DP values are representative for these fibre classes. For this reason, literature was reviewed and industry observations were collected to provide reference ranges indicative of the different textile classes. These findings are presented first, followed by the results of our measurements, which serve to illustrate and confirm the working of the toolbox.

#### Literature and industry observations

Raw cotton fibres typically exhibit a high DP, around 3000, though values from 2300 to 3000 have been reported depending on cotton type and growing conditions^30–32^. Similar values are found in fabrics; Palme et al., for instance, reported ~ 2900^29^ and 2621^33^ for two virgin linens.

Chemical treatments such as bleaching, common in textile processing, degrade cellulose chains, lowering DP. For *bleached virgin* cotton fabrics, DP values between 1600 and 2000 have been reported^34,35^.

Laundering and wearing further reduce the DP. Post-consumer textiles, having undergone extensive use and washing, therefore exhibit considerably lower DP values than pre-consumer textiles. Industrial laundering, as applied in the workwear and rental industries, has a particularly strong effect. Palme et al. report a reduction from ~ 2900 for new bed linens to ~ 1670 after 2–4 laundry cycles (−40%), ~ 975 after 50 cycles (−65%), and ~ 600 for discarded sheets laundered > 50–150 times and classified as end-of-life (−80%)^29^. Since it is common practice to wash linens 2–4 times before market introduction, a DP of ~ 1670 can be considered representative for pre-consumer.

Jasinska reported similar findings, with reductions of ~ 35–70% after 50 cycles. In this study, a reduction was observed from an initial DP value of 1675–2669 to 1100–1588 after 10 cycles and 789–1114 after 50 cycles^35^. These findings suggest that post-consumer textiles subjected to industrial laundering typically have a DP < 1600. Asaadi et al. likewise reported DP values of 1984, 1439 and 1022 for three ‘waste batches’ of hospital linens. The high-value batch may reflect discarding due to other factors such as stains rather than fibre degradation^36^.

The impact of industrial laundering is much stronger than that of domestic laundering. Wedin et al. found reductions of 70 to 80% after 50 industrial cycles, in line with other studies. In contrast, domestic laundering caused significantly smaller decreases: after 50 cycles, the DP of a cotton T-shirt fell from 2979 to 2277 (−24%) while a greige hospital bed sheet decreased from 7358 to 6581 (11%)^37^. Notably, the latter fabric had an unusually high initial DP. After 50 domestic cycles, degradation had not yet plateaued, suggesting further reduction with continued laundering.

Despite these studies, limited academic literature is available on the DP of post-consumer textiles as actually encountered in industry. Recycling partners report DP values below 1600 for post-consumer textiles, with no systematic difference between domestic and industrial sources. This is striking given the much stronger effect of industrial laundering observed in controlled studies. A likely explanation it that domestic use involves more cycles than typically investigated (i.e. >50), and that additional factors such as wear and sunlight exposure (UV-induced oxidative chain degradation) further lower the DP.

In practice, recycling batches are less homogeneous than controlled laboratory studies, with outliers both below 1000 and above 1600. These variations tend to average out, so typical DP values for post-consumer recycled material fall between 1000 and 1600. By contrast, pre-consumer recycled fibres – unused and minimally laundered – usually retain DP values > 1600. Exact values depend on cotton quality and prior treatments: for example, unbleached fabrics generally exhibit slighter higher DP-values.

For the proof of principle demonstration of the toolbox in this study, we assume DP-values < 1600 to indicate post-consumer recycled cotton fibres, and DP > 1600 to indicate pre-consumer fibres.

#### Measured DP-values

The DP of the textile test materials listed in Table 2 was determined, the results are presented in Table 5.

**Table 5:** DP-values as determined from the intrinsic viscosity measurements for the textile test materials (Sect. 3). Each sample was measured in duplicate, according to the method described in (Sect. 2. Methods.

**Name**	**Description**	**Mean value DP**		
		**Sample**		
		**1**	**2**	**3**
Fibre 1	Pre-consumer MR denim	1827	-	-
Fibre 2	Post-consumer MR cotton knitwear	1223	1385	-
Fibre 3	Lyocell RB	663	-	-
Fibre 4	Raw virgin cotton	3227	2427	-
Yarn 1	Polycotton yarn with pre-consumer MR denim	1661	-	-
Yarn 2	Polycotton yarn with post-consumer MR knitwear	1260	-	-
Yarn 3	Cellulose yarn with pre-consumer MR denim and lyocell RB	898	-	-
Yarn 4	Polycotton yarn with post-consumer MR knitwear and raw cotton	1962	1741	-
Fabric 1	Woven polycotton fabric with pre- and post-consumer MR fibre	1551	1826	*-*
Fabric 2	Woven polycotton fabric	1555	-	-
Fabric 3	Khaki polycotton woven fabric	1832	1794	1783
Fabric 4	White polycotton terry cloth	1893	1901	1890

For the cellulosic fibres (Fibres 1–4) the determined DP-values were consistent with their claimed origins (Table 2). For Fibre 1 (pre-consumer denim) a DP > 1600 was measured (1827), confirming pre-consumer origin. For Fibre 2 (post-consumer knitwear) DP-values < 1600 were measured (1223 and 1385), characteristic of post-consumer material. Fibre 3 (lyocell RB) yielded a DP of 663, matching values expected for lyocell^38^. For Fibre 4 (raw virgin cotton) DP-values of 3227 and 2427 were measured, consistent with untreated cotton^30,32^. The difference between the two samples illustrates batch inhomogeneity.

Next, Yarns 1–4 were analysed to validate the method’s ability to distinguish the origin of the mechanically recycled cotton. Yarn 1 and Yarn 2, both polycotton blends, contained either Fibre 1 (pre-consumer cotton) or Fibre 2 (post-consumer cotton). After polyester removal, DP values of 1661 and 1260 were measured, respectively, mirroring the fibre-level results taking into account batch inhomogeneity. These results demonstrate that, after separating polyester, the method reliably identifies whether the recycled cotton is pre- or post-consumer.

Yarn 3, composed of ~ 33% pre-consumer cotton (Fibre 1) and ~ 67% lyocell RB (Fibre 3), yielded a DP of ~ 900. This value corresponds to the weighted average of its components. However, due to the dominance of lyocell, distinguishing the origin of the cotton becomes more challenging.

Yarn 4 contained approximately equal fractions of polyester, mechanically recycled fibres (Fibre 2, post-consumer) and raw virgin cotton (Fibre 4). The measured DP-values (1962 and 1741) indicate the presence of post-consumer cotton. If only virgin and pre-consumer cotton were present, values > 2000 would be expected.

Finally, the DP of the fabrics was determined. Fabric 1 contained ~ 33% pre-consumer and ~ 67% post-consumer material. Two separate locations of the textile gave DP-values of 1551 and 1826. The lower value aligns within experimental error with the values of Fibres 1 and 2 and confirms post-consumer contribution. The higher value is higher than expected and likely reflects sampling of a premium cotton fraction. Because only milligram amounts are analysed, such outliers are possible in fabrics produced in a laboratory setting, especially when inhomogeneous recycled material is used. In industrial produced practice, large-scale fibre blending and homogenisation in blow rooms would minimize this effect. To improve reliability, future testing should use larger samples. Additionally, it would be advised to remove the yarns from the fabric and test the individual yarns separately, as is done in the fibre length distribution method, to increase certainty in the types and origins of cotton used.

Fabric 2, made from Yarn 3 (pre-consumer + lyocell RB) and Yarn 4 (post-consumer + raw virgin cotton) had a DP of 1555. This value fits with expectations from the fibre and yarn measurements, but illustrates the difficulty of interpretation when multiple cellulose sources are present, as the result represents an average.

Two industry-provided fabrics were also tested. When the full toolbox is applied, microscopy can be used to confirm the presence of *mechanically* recycled cotton. However, due to the exploratory nature of the study, here supplier information was used instead to confirm the presence of mechanically recycled cotton in Fabrics 3 and 4. The origin of the mechanically recycled cotton however, was not known.

Fabric 3, a khaki polycotton woven fabric, was labelled as containing 50% recycled polyester and 50% cotton-rich mechanically recycled fibres of mixed origin. For the three independent samples similar relatively high DP-values of ~ 1800 were found. This is a value typical for pre-consumer material and suggesting that post-consumer content is limited. Fabric 4 is a white terry cloth. For all three samples a DP-value of ~ 1900 was measured, indicating the cotton is likely of pre-consumer origin.

Overall, the results provide proof of principle: DP analysis can confirm the pre- or post-consumer origin of recycled cotton in (polycotton) textiles. The method performs reliably for single-fibre materials and simple blends, while interpretation is more complex for multi-cellulosic blends (Yarns 3–4, Fabrics 1–2), where the measured DP represents an average across the cellulosic fibre types. For example, a blend of virgin cotton and lyocell could yield a DP expected for post-consumer recycled cotton. Therefore, DP analysis should not be interpreted in isolation but in combination with the other tools in the analytical toolbox. In the end, the combination of microscopic images, fiber length distribution, and label information proves whether a blend of virgin cotton and lyocell or recycled cotton is present.

Nevertheless, the measurements show clear potential for DP analysis as a verification tool. Future work should focus on exploring methods to measure the polymer weight distribution and on mapping DP ranges across real-world recycling streams. A dedicated manuscript, in collaboration with industry partners, is currently in preparation on this topic.

## Summary and outlook

With new EU regulations – such as the EPR – facilitating the transition towards a circular economy, there is an increasing demand for test methods to independently and transparently verify “green claims” and counter greenwashing. This work describes the development of an analytical toolbox that ultimately will be able to semi-quantitatively analyse the (mechanically) recycled cotton content in textiles. Three methods were developed:

- *Microscopy*: a qualitative method capable of confirming the presence of mechanically recycled cotton fibres, based on the fraction of damaged and undamaged fibre ends observed. A large proportion of damaged fibre ends indicates of the presence of mechanically recycled fibres, whereas a high proportion of undamaged fibre ends is characteristic of virgin fibres.
- *Fibre length distribution*: a semi-quantitative method in which the fibre length distribution is measured and fitted, allowing the (mechanically recycled) content to be approximated.
- *Degree of polymerisation (DP)*: a method that reveals the pre- and/or post-consumer origin of mechanically recycled cotton.

By combining these methods, textile labels and sustainability claims made can be verified. The proof of principle of the toolbox was demonstrated by applying the three methods to in-house produced textiles with known compositions.

The toolbox can be further extended by readily available standard methods including the ISO 1833 fibre ratio tests – for determining non-cellulosic^22^ or non-polyester^39^ content – and (near-)infrared spectroscopy for identifying chemical fibre types (cotton, polyester, polyamide, viscose…)^17^. The toolbox can complement existing track-and-trace systems. Actual measured values and validated sustainability claims could be incorporated into digital product passports, further increasing the validity and reliability of such systems.

The ultimate aim is to implement the toolbox in test laboratories worldwide. To achieve this, the methods must be developed into proven standards, which requires further development and commercialization. For the microscopy method, future work will employ machine learning for automated image analysis and classification, and to establish quantitative threshold guidelines for the expected ratios of damaged and undamaged fibre ends in recycled and virgin fibres. Additionally, detailed studies will be conducted to measure DP-values of different recycling streams, and develop measuring DP *distributions* rather than a single average value. For the fibre length distribution method, improvements in sample preparation are needed to ensure an economically viable process, alongside the development of automated fitting algorithms. More broadly, guidelines on sample size and measurement frequency to ensure reliability, as well as investigations into the accuracy of the quantitative test methods, are required. As chemical recycling is expected to scale industrially for various feedstocks, test methods for chemically recycled fibres should also be developed.

Together, these efforts will ensure that the toolbox becomes a robust and widely accepted standard for verifying sustainability claims, supporting the advancement of textile recycling.

## Data Availability

The datasets used and/or analysed during the current study are available from the corresponding author on reasonable request.
